# Virtues as protective factors for adolescent mental health

**DOI:** 10.1111/jora.13004

**Published:** 2024-07-19

**Authors:** Shane McLoughlin, Kristján Kristjánsson

**Affiliations:** ^1^ Jubilee Centre for Character and Virtues University of Birmingham Birmingham UK

**Keywords:** character, mental health, virtues

## Abstract

This paper explores the decline in adolescent mental health and the weakening of traditional moral frameworks, positing education in the virtues as protective of mental health due to the intrinsic link between moral/existential wellbeing and psychological health. By integrating character education into school curricula, a continuous “dosage” of moral guidance may be an optimal way to ensure a gradual and ever‐clearer articulation of a life worth living and how to live well. The paper critiques popular clinical and positive psychological approaches to promoting wellbeing, which often miss the existential and moral dimensions of adolescent growth. The conclusion emphasizes the need for integrating moral education into mental health interventions to address the comprehensive existential and moral dimensions of adolescent development. This paper advocates for a proactive character developmental model that nurtures moral and existential growth, recognizing challenges with virtue and meta‐virtue development as integral to personal and moral evolution, and enhancing the moral and psychological fortitude of adolescents.

## INTRODUCTION

Adolescence is a critical developmental stage characterized by significant physiological, psychological, and social changes, which can be accompanied by the onset of mental health issues. In exploring these complexities, this paper first underscores the essential, yet often undervalued, role of moral considerations related to adolescent psychological development and mental health. We then argue that a deeper understanding of moral development is crucial for addressing the multifaceted challenges faced by young people today, affecting their wellbeing. Although clinical and positive psychology provide various essential interventions promoting wellbeing, we argue that they may not fully address the existential and moral dilemmas characteristic of adolescent growth. Clinical interventions often focus on episodic treatments after mental illness has occurred, and while positive psychology emphasizes the enhancement of virtues and character strengths, both may potentially miss the intricate ways in which virtues are expressed in everyday life.

Therefore, we advocate herein for a neo‐Aristotelian character developmental model, embedded in educational settings, which proactively nurtures moral and existential development. This paper explores how cultivating virtues through habituation contributes to psychological resilience, not by associating mental illness with moral failure but by acknowledging the normalcy of moral growth as part of adolescent development. Character education is proposed not as a critique of young people's character in the face of mental health challenges but as a supportive framework that recognizes such challenges as integral to personal and moral evolution. Our analysis elucidates how character education, far from reducing mental health issues to moral deficits, can offer a robust foundation for adolescents to build resilience around a moral compass and to navigate life's moral and psychological intricacies. In sum, we aim to articulate the profound impact that character education can have on mental health strategies, strengthening the moral and existential fortitude of adolescents.

## DECLINING MENTAL HEALTH TRENDS AMONG ADOLESCENTS

The issue of deteriorating mental health among adolescents has become a focal point of concern in recent years, attracting widespread attention from researchers, clinicians, and policymakers. In the United States, a comprehensive study by Keyes et al. (2019) offers invaluable insights into this decline. The study used data from the Monitoring the Future yearly cross‐sectional surveys, which encompassed a broad demographic of 8th, 10th, and 12th‐grade students. The study found a marked increase in depressive symptoms from 1991 to 2018. Notably, these symptoms initially decreased from 1991 until 2011 but then experienced a concerning resurgence, peaking in 2018. The longitudinal nature of this study, spanning over 28 years, provides a robust framework for understanding the temporal dynamics of adolescent mental health.

Research from the United Kingdom (Bitsko et al., 2022) corroborates the increasing prevalence of mental health issues among children and adolescents. Using data from 2013 to 2019, the study identified attention‐deficit/hyperactivity disorder and anxiety as the most prevalent disorders among U.K. children and adolescents aged 3–17 years. Alarmingly, among adolescents aged 12–17 years, approximately one in five had experienced a major depressive episode. These findings not only underscore the urgent need for immediate intervention and preventive measures but also highlight the critical role of public health surveillance in understanding the prevalence of specific mental disorders and other indicators of mental health.

The phenomenon of declining mental health among adolescents is not confined to the United States and the United Kingdom. A plethora of studies indicate that similar patterns are manifesting on a global scale. For instance, Dalsgaard et al. (2020) reported high rates of mental disorders diagnosed before the age of 18 in Denmark (14.63% in girls and 15.51% in boys). Moreover, a systematic review and meta‐analysis by Shorey et al. (2022) took a global perspective, examining the worldwide prevalence of depressive symptoms among adolescents. The study found that the Middle East, Africa, and Asia have the highest prevalence rates of depressive symptoms, thereby indicating that this is a global issue requiring concerted international efforts for mitigation.

While the overwhelming body of evidence suggests that mental health issues among adolescents are both escalating and ubiquitous, there are nuanced variations that warrant closer examination. Shorey et al. (2022) revealed that female adolescents had higher rates of depressive symptoms, adding another layer of complexity to the issue. A study by Rodway et al. (2020) delved even further into these complexities, finding that although girls reported experiencing various stressors like domestic violence and academic pressures more frequently, boys had significantly higher suicide rates. This was particularly pronounced among those aged 17–19. Xiao et al. (2022) go further with their meta‐analysis, revealing a high (>1 in 5) global occurrence of non‐suicidal self‐injury (NSSI) among adolescents in nonclinical samples. The study noted variations in the severity, methods, and underlying reasons for NSSI. Modern adolescents appear more prone to engage in repetitive NSSI behaviors, and the incidence of moderate to severe injuries is on the rise. Additionally, adolescents from single‐parent families or those with siblings are more likely to engage in NSSI, possibly due to difficulties adjusting to new family dynamics.

The declining mental health of adolescents is thus a pressing issue that has been substantiated by a multitude of studies from various countries. These studies collectively indicate that while mental health issues are universally prevalent among adolescents, the severity and type of these issues can vary based on factors such as age and gender. Therefore, a multi‐faceted, context‐sensitive approach that takes these variations into account is imperative for effective intervention and prevention.

This paper aims to fill a gap in the academic dialogue by intertwining existential struggles (e.g., reasons for living) with moral development. Eminent cultural critic David Brooks (2023) traces the mental health epidemic to the fact that we are enmeshed in “emotional, relational, and spiritual crisis,” undergirding “our political dysfunction and the general crisis of our democracy.” Philosopher Talbot Brewer (2023) similarly traces this development to the breakdown of the moral ecology that Aristotle, for one, saw as the essential backdrop for a healthy personal development, and its replacement with a culture of corporate profit‐seeking (e.g., by Big Tech), in which the “village” (that, as the proverb goes, is required to bring up a child) has been “all but shouldered out of its socializing role.” While we might use less dramatic words than Brewer for what he calls the current “tragedy of the cultural commons,” he and Brooks are right in pointing out the possible moral roots of some of the disturbing developments that have been described above. Brooks (2023) remarks dryly that “psychology is a wonderful profession, but its goal is mental health, not moral growth.” We follow upon that remark here by drawing upon sources outside of psychology. We begin by taking account of biological and economic variables affecting mental health that might be outside of practitioners' control. We then critically consider both popular and more controversial explanations for the observed decline in adolescent mental health. Finally, we argue for the importance of character education for taking a sustained and pre‐emptive approach to mental healthcare provision that we believe would offer a useful complementary solution to existing adolescent mental healthcare provisions. Unfortunately, the literatures on mental health and moral or characterological development have tended to run on parallel lines without many significant interactions (with a few exceptions such as Wee & de Broöns, 2018).

### Possible reasons for declining adolescent mental health

Various reasons have been proposed for the observed decline in adolescent mental health, such as biological maturation, and economic, technological, and social trends. In this section, we aim to give an overview of some of these considerations with a view to establishing probable contributors, along with commonly touted explanations that are lacking in evidence.

To set out our stall in a realistic way, we should first acknowledge that it has been well established for some time within mainstream science that there are biological underpinnings of many, if not most, mental health diagnoses. We know, for instance, that there are substantive changes in the adolescent brain with increases in white matter and cortical gray matter (Fuhrmann et al., 2015; Giedd et al., 1999). These increases are greatest in the frontal and parietal lobes (associated with intelligence; Jung & Haier, 2007) before age 12, allowing fuller comprehension of the complexities of life, including social dynamics, academic pressures, and existential questions (e.g., purpose in life; see also Gale et al., 2012). It is easy to suppose how cognitive overload may be overwhelming, contributing to stress, anxiety, or depressive symptoms, based on this factor alone. Moreover, their temporal lobes continue developing through age 16, which may be related to heightened emotional sensitivity or intensity. Puberty and hormonal change (Pfeifer & Allen, 2021) add to this further. Given the well‐known biological factors affecting mental health in general, and indeed in adolescence specifically, it is hardly surprising that there are also genetic associations with mental health (Anttila et al., 2018; Wray et al., 2018) and wellbeing (Baselmans et al., 2019), as these biological changes are largely a function of the genome itself. Finally, within applied fields such as clinical psychology, there are relatively fixed characteristics of individuals (e.g., personality) to which treatments might be tailored (Harkness & Lilienfeld, 1997). For example, some treatments might work better for introverts compared to extraverts. Therefore, those who might prefer environmental explanations for psychological phenomena, including educators, social scientists, and epidemiologists, face a complex uphill battle against a cascade of biological changes affecting mental health during the period of adolescence. Accounting for these factors, outside of practitioners' control, can help practitioners to cater for diverse needs and set realistic treatment expectations before turning to environmental causes of mental ill‐health. That said, the reasons for the recent changes in adolescent mental health are likely to be largely environmental.

Perhaps the most obvious factor that could promote or be a detriment to mental health, after factoring in our biological constitutions, are our material conditions. While not having basic needs met (e.g., food/shelter) is crucial to wellbeing, the picture is much more complex than this. For example, the genetic correlates of socioeconomic status (SES) are often stratified by region (Abdellaoui et al., 2019). Only some disease‐specific genetic variation has been separated out from genetic variation shared with SES (Marees et al., 2021). Therefore, the degree to which material conditions or genetic factors *cause* mental health problems is not fully clear; one may confound the other, or perhaps there could be a cyclical relationship between them. Nonetheless, if we can find substantial evidence to suggest that material conditions are at least *associated* with adolescent mental health, an obvious first step in treating these conditions would be to help to address it at the economic level. Some of the best predictors of suicidality are defeat, entrapment, and hopelessness (Dhingra et al., 2016; Oakey‐Frost et al., 2022; Taylor et al., 2011; Van Orden et al., 2010), and macroeconomic trends outside of the average person's immediate control are an obvious source of all three. Before we delve into more locally addressable and frequently touted factors, it is crucial to establish the foundational role of macroeconomic conditions. This contextualizes the often‐overstated impact of factors like social media, for which the evidence base is notably weaker than many might expect (see below). At the same time, highlighting material concerns that are salient in the context of adolescent mental health, beyond the control of mental health practitioners and educators, contextualizes the challenge ahead.

Arguably, the main reason currently touted for the rise of adolescent mental health problems is the rise of social media, which happened at about the same time. But to what extent does it account for it? There are several plausible reasons to believe that there are net negative effects of social media, such as a putative increase in cyber‐bullying, with largely ineffective interventions (Lan et al., 2022; Polanin et al., 2022), social reinforcement of certain disorders (e.g., “pro‐ana” communities support anorexia; Firkins et al., 2019), and generally a lower level of face‐to‐face interaction among adolescents. The literature on the putative detrimental effects of social media and smartphone use is quite mixed, however. Twenge (2020) argues that increases in depression, self‐harm, and suicide among U.S. adolescents are linked to their technology use. This is particularly pronounced in girls (Twenge et al., 2022). There is some evidence that personal smartphone use, in particular, increases the odds of adolescents experiencing depression, anxiety, higher perceived stress, and poorer sleep quality (Sohn et al., 2019). Other studies have found, however, that time spent on social media was not related to *changes* in depression or anxiety (Coyne et al., 2020), hinting at the possibility that one of the causes of social media use might be depression/anxiety in the first place, making the causal relationship between social media use and adolescent mental health unclear. Furthermore, other authors have conducted large‐scale reviews and found the effects of social media on adolescent wellbeing to be minimal (Orben & Przybylski, 2019). Finally, other studies have shown that, to some small degree, online spaces may provide sources of social support that could have salutary effects on adolescent mental health (Zhou & Cheng, 2022). While the rise of smartphones and social media has accompanied the observed decline in adolescent mental health since about 2010, on aggregate this relationship is not especially strong, and large‐scale review studies have cast some doubt on the degree to which social media *causes* the observed decline in adolescent mental health. While undoubtedly important to research, it is becoming ever clearer that the trends in which we see increased demoralization, suicidality, and susceptibility to mental illness are not explained by the rise of social media to the degree that many might presume. This forces us to look outside to consider other macro‐environmental trends.

#### The decline of traditional morality anchors

In the past several years, we have also witnessed an erosion of the influence of various ethical institutions over time. Returning to the earlier insights from Brooks (2023) and Brewer (2023), we outline key social institutions that have traditionally had an important influence on what is considered to be ethical, which have changed quite radically in a short space of time.

In the United Kingdom, the British Social Attitudes Survey has found that more people have reported having no religion, while fewer people have identified as Christian over time (Curtice et al., 2019). The Pew Research Centre (2022) and Gallup Polls (2022) have found a similar pattern in the USA. We do not wish to take a stance on the value of particular religions, nor claim that different religions are equally ethical, or indeed to suggest that all tenets of any given religion are ethical. To complicate matters, several of the world's most influential “spiritual” traditions (such as Confucianism, Buddhism, and Ubuntu) refuse the label of “religion.” However, it is undeniable that, historically, religious and spiritual institutions have functioned to calibrate societies' moral compasses. If notions of God or some other transcendent power (be it theistic, pantheistic, or spiritual without being religiously tethered) are replaced by “golden calf” alternatives such as amoral ideologies, celebrity worship, populist beliefs, tribalism, “power” as such (e.g., as defined in postmodernism), commercial assets, or other amoral tele, this constitutes a radical deviation from the ethical cornerstones of society within the blink of an eye, in evolutionary terms. This is potentially important as religiosity is positively related to mental health (Levin & Chatters, 1998; Manoiu et al., 2023), and this relationship does appear to be causal (Garssen et al., 2021). While “religion” and “spirituality” are broad categories where there is much nuanced discussion to be had, these findings do give us some initial indication of a positive link between personal ethics and mental health.

Another major cultural shift that for most of our evolutionary history has been a major source of moral foundations is the dissolution of the traditional family unit. In the USA, for example, marriage rates are in decline (Jones, 2020). Similarly, divorce rates are extremely high compared to historical norms and increasingly being seen as morally acceptable (Dugan, 2017). There are data to suggest that, in various ways, divorce and some non‐traditional family structures relate to rates of juvenile delinquency (Burt et al., 2008), poor mental health (Ashworth et al., 2022; D'Onofrio & Emery, 2019), and indeed, may affect moral development (Padilla‐Walker & Memmott‐Elison, 2020). It is important to note that the aim here is not to pass moral judgment on changing family structures, but rather to highlight their potential unforeseen implications for wellbeing on aggregate at a population level (i.e., of course this will not apply in all individual cases), given the rapidity with which this shift occurred relative to the amount of time our species has existed. The shift in family dynamics is a complex phenomenon influenced by a myriad of factors, and it is crucial to explore its impact in a nuanced manner.

## MORALITY IN CLINICAL PSYCHOLOGY

The decline of traditional moral anchors in society, coupled with the increasing prevalence of adolescent mental health issues, necessitates an investigation into the moral dimension of mental health support. As cultural and societal norms have evolved rapidly, leading to ambiguity regarding the moral foundations of culture, a tangible impact is arguably seen on adolescent mental health. These shifts contribute to a milieu of existential uncertainty that compounds pre‐existing mental health challenges. It is therefore imperative to integrate moral considerations into the fabric of mental health interventions, particularly for younger populations who are in the formative stages of their moral development and identity formation.

Many fields weigh in on the topic of mental health and wellbeing, but clinical psychology arguably carries special weight in the conversation, given over a century of empirical insights to be gleaned. Clinical psychology operates historically on the foundational implicit premise that an individual has not yet reached their fullest potential. The clinician's role is not merely to diagnose and treat but to facilitate a person's journey toward becoming the best possible version of themselves. This involves helping the individual to articulate areas of shortfall and collaboratively devising a plan for improvement. Although commonly under‐acknowledged, this endeavor is not purely scientific but is deeply rooted in existential and moral concerns, reflecting the complexities of human experience. Indeed, moral “reasons for living” are generally protective of suicidal thoughts and behaviors (Bakhiyi et al., 2016; Linehan et al., 1983), while having a clear articulation of one's values is associated with young peoples' wellbeing, purpose in life, and supports autonomous functioning (McLoughlin et al., 2022). Moreover, having an ideal moral self is associated with fewer internalizing and externalizing behaviors (Hardy et al., 2014); that is, pushing one's feelings down and withdrawing, or on the other hand, outward displays of emotional volatility.

Numerous therapeutic approaches incorporate values or virtues in different ways to guide treatment. Logotherapy, developed by Viktor Frankl, centers around the existential search for meaning (Martínez & Flórez, 2015). Frankl posits that the primary human drive is not pleasure, as suggested by Freud, but the pursuit of what we find meaningful. It is value‐oriented in that it encourages individuals to identify what truly matters to them, thereby serving as a compass that directs all other behaviors and decisions. The therapy aims to help patients find meaning even in suffering and despair, thereby creating a moral dimension to the therapeutic journey. Acceptance and Commitment Therapy (ACT) places a significant emphasis on value‐guided actions (Bramwell & Richardson, 2018). ACT encourages individuals to identify their core values in various life domains such as family, career, and personal growth. The focus is on aligning actions with these values, even when faced with psychological obstacles like negative thoughts and emotions. By acting according to one's values (e.g., staying socially engaged despite being ostracized; Hochard et al., 2021), the individual moves toward a life that is more fulfilling and, ideally, experiences less psychological distress. Compassion‐Focused Therapy (CFT) is another approach that integrates values, specifically the value of compassion, both toward oneself and others (Gilbert, 2014). CFT posits that self‐compassion and compassion for others are essential components for mental wellbeing. It not only addresses emotional pain but also provides a framework for ethical considerations and moral actions through the cultivation of compassion. Behavioral Activation Therapy (BAT) directs focus on values by helping individuals understand the connection between their behavior, emotions, and values. It assists in identifying life areas that are most important and aids in aligning daily activities and actions with those valued life areas. The central tenet is that by behaving in line with one's values, one can increase life‐satisfaction and decrease symptoms of depression (Sturmey, 2009). Dialectical Behavior Therapy (DBT) also incorporates values, albeit indirectly, through its emphasis on mindfulness and acceptance skills. One of the core modules in DBT is “emotion regulation,” where individuals are taught to identify and label their emotions and to act in ways that are consistent with their long‐term goals and values (see Cameron et al., 2014). Cognitive Behavioral Therapy (CBT), perhaps one of the most widely practiced and effective therapies (Butler et al., 2006), is less overtly value‐focused than some other approaches, despite its historical roots in the values of Stoicism. However, it does involve the examination of underlying beliefs, which often include personal values and morals. By challenging distorted thought patterns and beliefs, the aim is often to align an individual's cognitive schema more closely with their core values, even if this is not explicitly stated.

Despite the attention given across psychotherapies to important moral topics such as values, meaning, and so on, there are important philosophical and practical challenges to address. A key philosophical challenge facing values‐focused therapies is the nature of values themselves; not all endorsed values are universally beneficial or benign (McLoughlin & Roche, 2022; Ruiz & Roche, 2007). Morally worthy values that are ingrained in an agent's characterological make‐up (i.e., as *virtues*) are worth pursuing in values‐focused psychotherapies, whereas there are other values a client could hold that may not be conducive to the flourishing of clients or those they interact with. In some values‐focused psychotherapies, it is argued that other people might regulate which values are acceptable to hold (see Barnes‐Holmes et al., 2000). However, history offers countless examples of collective malevolence sanctioned by social agreement, underscoring the dangers of delegating the regulation of individual values to external entities like groups or institutions. Therefore, a focus on morally worthy values (e.g., moral and civic virtues) based on some objective criterion remains, in our view, the optimal option.

Another perhaps more practical challenge faced is that, while clinical psychology directly tackles existential issues like anxiety and depression, it also faces operational bottlenecks that limit its effectiveness. Within publicly funded health systems like the NHS, bureaucratic delays can result in long waiting times for those seeking mental health support. In private healthcare settings, the cost of therapy can be prohibitively high. The standard 30‐ to 60‐min weekly sessions (NHS, 2021) may also be insufficient for many individuals grappling with complex, deep‐seated existential issues relating to abstract ideas such as their purpose in life.

Clinical psychology has also been accused of being fundamentally reactive, aimed at treating conditions after they manifest, rather than preventive (Seligman, 2019). Adding to these challenges is the fact that many therapists have limited research training, given the heavy focus on practical experience in their education, compromising their ability to critically evaluate the latest therapeutic methodologies beyond the potentially biased qualitative experience they or their mentors may hold (Lilienfeld et al., 2013). This situation is exacerbated by the financial incentives for psychotherapy researchers, who may gain notoriety and income from “selling” a particular therapy, often without robust empirical evidence to support its efficacy. Indeed, meta‐analyses have shown that researcher allegiance inflates therapeutic outcomes within randomized controlled trials (Munder et al., 2013).

It is clear that a significant proportion of individuals seeking clinical psychological support are grappling with existential and moral issues that would ideally be addressed much earlier in life. Schools offer a consistent and structured environment for proactive character development, aimed at instilling virtues and ethical principles that can help forestalling the development of mental health issues later on. The daily interactions and experiences in the educational setting provide ample opportunities for moral cultivation, thereby acting as a form of preventative mental health care. In its current form, clinical psychology is more of a palliative measure, intervening only after problems have manifested, rather than a holistic solution that integrates preventative aspects. The focus needs to shift toward a more comprehensive approach to mental health, which includes moral development as a foundational component. We now turn to more viable options: Positive education and Neo‐Aristotelian character education.

## POSITIVE EDUCATION?

“Positive education” is typically understood as the educational incarnation of positive psychology, which came into prominence at the beginning of this century. At the heart of positive psychological theory lies a fundamental shift from a deficit‐focused to a strength‐based paradigm. Where traditional psychology has often been preoccupied with pathology and remediation, positive psychology (and, by implication, positive education, see Seligman et al., 2009) posits a broader perspective, one which highlights the pursuit of overall wellbeing (both subjective *qua* happiness and objective *qua* flourishing) and the cultivation of virtues (Seligman, 2011). It is a framework that resonates particularly with the developmental stages of adolescence, a period marked by rapid growth, exploration, and identity formation. The underlying assumptions of this approach are rooted in the belief that individuals not only seek to alleviate suffering but also aspire to thrive, to harness their innate potential and to forge meaningful lives. Within this perspective, the building blocks of wellbeing are character strengths and virtues—qualities that enable individuals and communities to flourish. These ingredients, integral to the fabric of positive development, provide a scaffolding for interventions aimed at nurturing resilience, fostering engagement, and encouraging a sense of purpose among young people.

The lexicon of positive psychology is enriched with terms such as “flourishing” and “thriving,” which capture the essence of what it means to lead a full and meaningful life (Seligman, 2011). This emphasis on positive outcomes necessitates a deeper exploration of the role played by character strengths and virtues. As cornerstones of positive psychological interventions, these qualities are woven into educational programs, therapeutic strategies, and community initiatives designed to bolster the psychological capital of young individuals. Such interventions often involve the development of self‐awareness, the nurturing of empathy, and the encouragement of perseverance, providing adolescents with the tools to navigate life's challenges. Whether it is through mentorship schemes that foster wisdom and leadership or through curricula that integrate practices of gratitude and mindfulness, the aim is to cultivate an environment where young people can not only identify but also apply their strengths in ways that contribute to their wellbeing and that of those around them.

### Relevance and advantages for studies of adolescence

There are many things to like about positive psychology and positive education, not least in the context of exploring ebbs and flows in the lives of young people. Departing from the medicalized model that often characterizes clinical psychology's approach to the ills that can befall individuals and prevent them from reaching their developmental potential, positive psychology mediates a marriage between “business‐as‐usual” psychology and the development of moral purpose of the kind explained in the preceding section. It does so by (a) shifting the focus in psychology from negative variables—deficits and disorders—to positive, flourishing‐inducing ones, and (b) translating some of the basic tenets from philosophy on what it means to live a “good” life into empirically viable terms, issuing in a psychology that acts as the “social science equivalent” of virtue ethics (Peterson & Seligman, 2004, p. 89).

The biggest contribution of positive psychology to the concerns motivating the present paper is its theory of six overarching virtues and 24 underlying character strengths. While various (see the next sub‐section) misgivings abound, there is no denying the fact that it has revolutionized approaches to character strengths and virtues within psychology, mainstream as well as “positive,” and beyond, not least simply by putting them on the agenda of studies of adolescent—and adult—wellbeing. For example, this paper would probably never have been written if not because of Peterson and Seligman's (2004) work, as there was little appetite for considerations of this kind predating their work within social science (as distinct from pure philosophy). Moreover, there is ample empirical support for the cross‐sectional relationships between positive psychological virtues and wellbeing (Azañedo et al., 2021; Wagner et al., 2020), including in adolescence (Gillham et al., 2011). Indeed, multi‐component positive psychological interventions appear to improve adolescents' wellbeing (Tejada‐Gallardo et al., 2020), though most of these studies have some risk of bias.

The 24 strengths in the Values‐in‐Action (VIA) model (Peterson & Seligman, 2004) include a mixture of moral, intellectual, performative, and (to a significantly lesser degree) civic virtues or sub‐virtues. Given their background in mainstream psychology, most positive psychologists appear to be more comfortable talking about performance virtues than moral or civic ones (see, e.g., Duckworth, 2016, on grit). That is not necessarily a bad thing, as what holds many young people back from acting morally may not be a lack of moral virtues as such but rather a lack of psychological skills to put those into practice. Yet the emphasis on performance virtues or skills arguably does not get at the moral/existential factors (e.g., the development of moral identity) that are missing from contemporary clinical psychology.

The second contribution of positive psychology is moving the dial of wellbeing studies further toward the construct of flourishing. Seligman (2002) originally proposed a hybrid view of wellbeing, but with the center of gravity leaning heavily toward subjective (life‐satisfaction) rather than objective variables. In 2011, however, Seligman published his landmark book, *Flourish*, in which the goal of positive psychology is put forward as wellbeing *qua* flourishing. Seligman devised a list for the actual “elements” of wellbeing, understood as flourishing: “positive emotion, engagement, meaning, positive relationships, and accomplishments” (i.e., PERMA; 2011, p. 16). In this new account, universally valued virtues (Peterson & Seligman, 2004) play an even more prominent role than in the 2002 account; they now undergird all the PERMA‐elements (2011, p. 24). Despite the emphasis on objective elements in his new account, Seligman acknowledges that one of them—positive emotion—is subjective and that the others incorporate some subjective components. He thus prefers to refer to his approach as pluralistic rather than objective. Nevertheless, Seligman now considers all the elements of flourishing to be underpinned by objective moral virtues. This is a hugely important move, because it shifts the initial focus of any viable studies of adolescent wellbeing—or the lack thereof—from internal psychological factors to factors that have to do with their objective flourishing.

Thirdly, positive psychology adds a welcomed universalist flavor to studies of adolescent wellbeing. In the psychological field, which has since its inception been shy of making universalist claims (with a few notable exceptions, such as Kohlberg), because of its commitment to non‐value‐ladenness, it is refreshing to witness a theory that promotes the universality of the character strengths and virtues, and does so on grounds of empirical research (however incomplete), rather than mere philosophical stipulation (McGrath, 2015). While universalism of this type does not imply that teenage floundering cannot have different causes in different cultures and at different times, positive psychological researchers will apply the same overall lens to study adolescent flourishing wherever they go, knowing that empirical evidence has shown that the same character strengths predict flourishing universally.

### Weaknesses in positive psychological theory

The above sub‐section may have created the impression that positive psychology has found the ideal middle point between clinical psychology and traditional philosophical character theory, by synthesizing insights from both. While there is some truth to that, in our view, we will not shirk here from presenting some significant weaknesses in the positive psychological arsenal, especially if viewed from the perspective of neo‐Aristotelianism (see responses to some of the below in McGrath, 2019).

First, compared to philosophical accounts, grounded in a universalist, realist ontology of value (i.e., an ontology that sees values as objectively grounded in facts about the world in which we live), the universalism lauded above seems to rest on an essentially infirm foundation. Peterson and Seligman (2004) stress repeatedly that theirs is not a theory of what is *universally valuable* but just what happens to be *universally valued*. Hence, one might argue that positive psychology/education runs the risk of focusing in on arbitrary values that people may hold, which may or may not be true and which may or may not be moral.

Second, positive psychology does not impose any golden‐mean architectonic on its virtues and character strengths; rather, more of each virtue is considered better. However, this imports various problems, both theoretical and practical. Theoretically, the idea that extreme exemplifications of a virtue cannot turn into vices creates some notable philosophical and psychological paradoxes (Grant & Schwartz, 2011; Ng & Tay, 2020). Practically, by indiscriminately trying to instill a virtue of, say, gratitude in adolescent students, the teacher can create excessive educational forms of the virtue: say, in the case of gratitude a Stockholm syndrome where the students learn to be uncritically grateful for whatever treatment they receive (Morgan et al., 2015). This uncritical amplification of virtue, if naively correlated with mental health, might imply that those experiencing mental health issues simply lack certain virtues, thus painting a picture of moral deficiency. It could also suggest that to be mentally unwell is, in some sense, to be less virtuous, rather than not knowing which virtues to act on in a context‐sensitive, practically wise manner (Kristjánsson & Fowers, 2024). This is a deeply problematic stance, as it overlooks the complex interplay between character strengths, mental health, and situational factors. Instead, a nuanced understanding of virtues acknowledges that moral excellence is not merely the presence of virtues in abundance but their judicious application, tempered by practical wisdom. From this vantage point, we see mental health challenges not as an indictment of one's moral character but potentially as indicative of areas where the habitual exercise of moral discernment has yet to mature. Such a view fosters a more empathetic perspective on mental health struggles, seeing them as part of the human experience where moral understanding—and not moral substance—is still developing. By aligning character education with this philosophy, we can support adolescents in cultivating not only the virtues themselves but also the wisdom to apply them appropriately, thus contributing to a holistic approach to mental health that is free from any overly moralistic judgment.

The third problem has to do with the lack of any intellectual virtue that acts as the moral integrator in the VIA‐system. What sort of decision procedure can an adolescent activate when faced with a conflict between, say, honesty to teachers and loyalty to friends? Approaching this concern from a positive psychological perspective, McGrath (2019) suggests that the role that historical virtue theories ascribe here to *phronesis* or practical wisdom (Kristjánsson & Fowers, 2024) can be played collectively by three of the 24 VIA character strengths: prudence (for the assumed regulative function of *phronesis*), judgment (for the assumed constitutive function), and perspective (for the assumed integrative function). However, we worry that if the metacognitive role standardly ascribed to *phronesis* is to be taken over by three discrete virtues at the same epistemological and characterological level, how will conflicts between the requirements of judgment, perspective, and prudence be adjudicated? Which of the virtues calls the shots—and why? Character‐related factors causally implicated in improving wellbeing appear to go beyond the use of individual virtues, and focus more on the central factors that orient and adjudicate between virtues, such as orientation toward “the good” (Weziak‐Bialowolska et al., 2021). While there is ample evidence that positive psychological virtues are at least *associated* with wellbeing (Blanchard et al., 2020; Hui et al., 2020; Portocarrero et al., 2020), and interventions *aimed at* improving virtues increase wellbeing to a small degree (Carr et al., 2021; Koydemir et al., 2021), it is unclear why. For example, it could be the case that improving one's character strengths means being more desirable to others because of those specific strengths they value (Niemiec, 2023), or that improving individual strengths along with other antecedents aggregate toward an improved vision of one's best moral self, used to regulate virtue expression (Hertz & Krettenauer, 2016; Krettenauer & Stichter, 2023; Xu et al., 2023).

In sum, we worry that the system of character strengths produced by positive psychology—undertheorized as it seems to be—is too eclectic to serve as the essential anchor for young people's decision‐making. Despite the emphasis on character strengths and virtues, the focus tends to be directed at the performative and instrumental rather than the moral and intrinsically valuable, and positive psychology seems overly cavalier about virtue conflicts, which we would argue are often the cause of existential crises in adolescence. That said, any psychology of adolescent wellbeing that fails to take seriously some of the best insights from positive psychology and positive education must count as inadequate in today's academic world.

## NEO‐ARISTOTELIAN CHARACTER EDUCATION

The weaknesses in positive psychology/education identified above raise the question if we would perhaps be better served by reviving the ancient (in particular, Aristotelian) account of character development on which positive psychology claims to partially draw. While it would be completely wrong—indeed, a “category mistake”—to consider the recently rejuvenated neo‐Aristotelian theory of character development and education (Fowers et al., 2021; Kristjánsson, 2015; Wright et al., 2020) as a replacement for clinical psychology in relation to adolescents' mental wellbeing, we explore various reasons in this section for why this theory adds value to the discursive field (see further in Peterson & Kristjánsson, 2024).

Neo‐Aristotelian character education (Jubilee Centre, 2022), understood here as the practical application of neo‐Aristotelian virtue ethics (Annas, 2011), is most often understood as an approach to valuing and cultivating character and virtues (similarly to Peterson & Seligman's, 2004, positive psychological approach). However, the grounding concept of character education on a (neo‐)Aristotelian understanding is actually not character or virtue but rather *human flourishing*—of which positive character traits are seen as constitutive parts, but by no means the only parts. For while we justify character traits in terms of their conduciveness to flourishing, we do not justify flourishing in terms of how it contributes to good character, but rather it is constitutive of the good life for individuals and societies (Aristotle, 1985, Book 1).

Flourishing accounts, such as Aristotle's, form one of the two main types of wellbeing accounts. Happiness accounts understand the criteria of wellbeing to be subjective in the sense of referring to psychological states (experiences, attitudes, feelings, beliefs) of an agent. Flourishing accounts, however, belong to objective accounts, according to which the criteria of wellbeing have to do principally with objective features of the agent—facts about her life—that can, in principle, be viewed from the outside. That said, to flourish or live well in the distinctive human way will give us happiness, or a sense of satisfaction, as an “ornament,” because it is part of human psychology to enjoy the exercise of our realized capabilities.

*Character*, in this theory, is understood as the reason‐responsive, evaluable, and educable part of human personhood or selfhood. It comprises, inter alia, virtues and vices, and it is obviously the former that character educators are interested in cultivating. *Virtue* (of character) is specified as (a) a relatively stable state of character that helps us decide what to do, (b) consisting in a golden mean, (c) relative to the individual, (d) which is defined by reference to how the practically wise person would understand it. It is a golden mean between two vices/extremes, one of excess and one of deficiency: for instance, bravery is a mean between cowardice and foolhardiness (Aristotle, 1985, p. 44 [1106b36‐1107a3]). Contemporary character educationists typically delineate four kinds of virtues: *moral*, *intellectual*, *civic*, and *performative* (Jubilee Centre, 2022). One of the intellectual virtues, practical wisdom (*phronesis*), is particularly relevant for the topic of this paper, and we return to it in the following sub‐section.

Neo‐Aristotelian character theory has many advantages, especially having to do with the clarity of its theoretical assumptions. Those include a realist *moral ontology*, according to which moral discourse describes an objective world of evaluation rather than simply evaluating subjectively an objective world of facts, and a *character‐educational theory*, according to which each stage of life requires different approaches for the development of character, beginning in childhood with habituation and the emulation of role models.

### Relevance for adolescence

Neo‐Aristotelian character theory offers a broad, holistic view of the development of reason‐responsive character traits in young people. Aristotle was acutely sensitive to the various factors, external and internal, that can prevent flourishing—as an ongoing activity—from happening. Although he had nothing specific to say about young people's mental health issues—as there was no academic discourse on this topic at his time—he offered a very extensive account of various possible causes of a lack of flourishing. His flourishing‐and‐character theory was based on an ideal of psycho‐moral and psycho‐social *homeostasis*. In Aristotle's teleological system, the parts of the soul are arranged such that they can adjust successfully to the various social situations in which individuals find themselves (inter alia, by adopting medial states of character). Whatever the merits or demerits of Aristotle's primitive biology of human beings, what stands out is the focus on (a) the inter‐connectedness of the challenges that complicate the task of growing‐up well and (b) the impossibility of separating health‐related challenges from moral and existential ones. The big challenge facing adolescents, in Aristotle's view, would be to form the right opinions on, and the proper emotion‐driven attitudes toward, the *telos* of their lives (their moral identity) and to learn to live those out consistently and coherently in their decision‐making.

The homeostasis in which Aristotle was interested relates to the identification of the golden mean as the ideal equilibrium for each individual virtue, but also to the harmonization and integration of the individual virtues, especially in circumstances where those seem to come into conflict with one another (e.g., where loyalty to friends clashes with honesty to teachers or parents). For Aristotle, the biggest developmental task of adolescence and early adulthood is thus the cultivation of the intellectual virtue of practical wisdom (*phronesis*), which serves this integrative function and various related ones (Kristjánsson & Fowers, 2024).

The greatest practical value of neo‐Aristotelian character theory for adolescence is probably its nuanced account of how young people's flourishing can be nourished through “caught,” “taught,” and “sought” methods of character development (Jubilee Centre, 2022). Aristotelians tend to highlight the importance of “catching” the right habits from the environment/ethos (family, school, neighborhood) at an early age, to set the stage for mature individual decision‐making at a later developmental stage. Indeed, Aristotle had nothing but scorn for those character warriors who believe that if you just make a personal effort to be good, you will automatically flourish. Even in adolescence and adulthood, when you have become capable of deliberation and reflection, Aristotle thought that the best way to ensure that you decide on the right things for the right reasons is through sustained critical engagement with your best friends and mentors.

Aristotle's texts give considerable detailed guidance to educators about how to “teach” moral character to children and young adolescents. However, for some reason those texts remain mostly mute on the best teaching methods that work in late adolescence and beyond—perhaps because of Aristotle's sustained, but mostly implicit, focus on the value of friendships as a “method” of moral development at that age. Most importantly, for this age group, Aristotle shifts from caught and taught methods to what we could call “character sought”: namely, the need for teenagers and young adults to venture beyond the habits with which they were inculcated in childhood, by subjecting those to their own critical scrutiny, thus seeking what we would nowadays designate as their “moral identity,” or their blueprint of a flourishing life. Evidently, Aristotle was not responding to any epidemic of mental ill health among adolescents at his time—and the varieties of psychological disorders that can afflict young people were mostly unknown to him. However, to couch this in modern language, Aristotle understood the developmental task of adolescence to be about *moral identity formation*, and his diagnosis of a crisis among young people at any given time in history would probably seek out, first and foremost, possible socio‐moral causes for the breakdown of successful formation of that kind, such as those raised in the previous section. Having a clear blueprint of a flourishing life that imbues adolescents with moral worth, including from their own perspective, is crucial for maintaining mental health. This moral blueprint can prepare young people to derive meaning and purpose in a world which not long ago felt far away: one in which it is becoming increasingly apparent that, for instance, innovations in AI will render many current jobs redundant (Kelly, 2023), thus possibly compromising the role of work as a major source of meaning and purpose at present (Romney et al., 2024).

### Weaknesses in neo‐Aristotelian theory

Motivated by Aristotle's own methodological naturalism, neo‐Aristotelians tend to revise and update Aristotle's account in light of the latest psychological evidence. However, there are some weaknesses in his theorizing that require more radical overhaul than just tinkering at the edges. First, Aristotle did not believe that humans are endowed with any genetically determined moral concerns, such as empathy; those were for him the products of upbringing. Many modern psychologists would disagree (Hoffman, 2001). Second is Aristotle's early‐years determinism: his contentious belief that if you are not brought up in good habits in early childhood, you will never see the moral light later (critiqued in Kristjánsson, 2015, chap. 5). Third is the puzzle of how a transition can take place between purely heteronomously inculcated traits in childhood and personally/critically chosen traits in adolescence. Fourth is Aristotle's radically down‐to‐earth “disenchantedness.” There is no emotional virtue of awe in his system and he had no place for epiphanic searches for transcendent values. This would arguably have prevented him from understanding adolescents' attraction to “out‐of‐this‐world” experiences, often aspired to through experimentation with drugs and other risk‐taking behaviors. Fifth, Aristotle was understandably not aware of the Enlightenment ideals of authenticity and autonomy; and despite his emphasis on the necessary individualization of virtue, he would have found it difficult to grasp the idea that a teenager could find her autonomously arrived‐at, authentic identity just by concentrating on one set of values, however important (say, the values of environmental protection). Moreover, late‐postmodern “identity politics,” with their inherent subjectivization of value, would for better or for worse, have been completely alien to him.

All in all, while it is far from being the case that either traditional Aristotelian or empirically updated neo‐Aristotelian character theory offers a panacea for current worries about the deterioration of adolescent wellbeing, those theories provide a complementary viewpoint to that of clinical psychology. Dimly, if at all aware of many of the bio‐environmental factors that clinical psychologists pay attention to, Aristotle would have reminded us not to forget civic and characterological variables that also play a large role in the lives of young people and can even determine whether they flourish or flounder.

## CHARACTER EDUCATION AND MENTAL HEALTH: MOST IMMEDIATE EMPIRICAL LACUNAE

The theoretical merits of neo‐Aristotelian character education notwithstanding, accurate and reliable measurement is fundamental to transforming character education from a philosophical concept into an evidence‐based practice that might be used to protect against mental health issues and promote flourishing. That is, if we cannot “measure” character, we cannot make any empirical claims about its relationship to anything else of interest, such as wellbeing. Robust psychometric validation provides empirical grounding for the very existence of character strengths, which are psychological characteristics, even if often proposed by philosophers, historians, or theologians. Consider for a moment that a short measure of Grit (Duckworth & Quinn, 2009), a popular putative performance virtue from the early positive psychology movement, accidentally measured the amoral personality dimension of conscientiousness (Ponnock et al., 2020). The implications of this, given that this measure (alone) had been cited thousands of times, are that thousands of studies may have drawn under‐supported conclusions. Separating character from other established constructs is crucial for ensuring that character education focuses on morally relevant attributes rather than broader temperamental qualities, which we already know relate to mental health (Bucher et al., 2019).

To advance character education as an empirical flourishing and prevention science, an essential early step is to develop standardized, norm‐referenced tests specifically tailored for the adolescent population. By establishing reliable developmental benchmarks for character development, these assessments enable educators and psychologists to quantitatively gauge the effectiveness of character education interventions and make informed decisions based on individual developmental needs (Wright et al., 2020). Some such measures exist, such as the Defining Issues Test (Rest, 1975) and the Intermediate Concept Measure (Thoma et al., 2017), but these tests come with their own problems. For example, the Defining Issues Test scores test takers' reasoning within moral dilemmas in relation to neo‐Kohlbergian theory, for which empirical support is less than unanimous (Kay, 1982). The Intermediate Concept Measure also presents moral dilemmas, with a key difference being that test takers are scored against what a group of designated experts might agree is the correct answer. Both measures are quite time‐intensive, meaning that it is not possible to assess an individual across a wide range of scenarios such that inferences can be made about the person's general character—the latter being crucial for ensuring predictive validity across novel scenarios, which is why we might want to measure character at all. Instead, norm‐referenced measures that allow an individual's score to be interpreted relative to others their age are likely to be more educationally informative, providing insights into relative moral standing. Other measures abound such as the VIA for Youth (Han, 2019) and the Moral Character Questionnaire (Michael Furr et al., 2022), but these are not sensitive to, for example, vices of excess or the role of *phronesis*.

Exploring the relationship between character traits and flourishing is another vital area of inquiry. While there is some reason to believe that virtue‐based reasoning at school can lead to flourishing in later life (McLoughlin et al., 2023), it is also essential to determine whether character as such correlates with overall wellbeing in a linear fashion, which would suggest “more is better” (*qua* positive psychology), or if they follow a non‐linear pattern such as the inverted U‐shape proposed by Aristotle's golden mean. This investigation not only tests philosophical theories empirically but also guides the development of character education practices that aim for balanced virtue expression in service of young people's wellbeing.

Finally, determining which moral theories most effectively contribute to an understanding of adolescent mental health and flourishing requires careful empirical scrutiny. Not all moral theories hold equal weight when translated into practical measures for mental health outcomes, making it essential to evaluate each theory not only on its philosophical merits but also on its empirical utility. For instance, positive psychology provides a robust framework for operationalizing constructs like wellbeing and resilience that are directly measurable and align well with educational programs; yet it often fails to capture the moral aspects of adolescents' identity formation and potential existential struggles. Neo‐Aristotelian Virtue Ethics, though rich in philosophical tradition, may offer concepts like *phronesis* (practical wisdom) that are more challenging to measure and implement within a school setting (Kristjánsson & Fowers, 2024). Empirical evaluations that measure the correlation between these theoretical constructs and indicators of flourishing—such as psychological wellbeing, social integration, and academic success—can provide critical insights. These studies must be rigorously designed to ensure that they not only capture the essence of each moral theory but also reflect their practical applicability in diverse educational environments. By identifying which theories most accurately predict mental health outcomes, educators and psychologists can better tailor character education programs that are both philosophically sound (i.e., they make sense conceptually) and empirically validated (i.e., they actually work), ensuring that they effectively support the moral and mental development of adolescents.

## CONCLUSION

Understanding the interplay between character education and mental health necessitates a clear delineation between the concepts of mental illness and morality. Mental illnesses, which can range from mood disorders to psychoses, are not indicative of an individual's moral character. They are complex conditions that can arise from a confluence of genetic, biological, environmental, and psychological factors. To conflate mental illness with immorality would be a grave and stigmatizing error. It is crucial to articulate that character education, while it may indirectly benefit mental health, does not impose a moral judgment on those who experience mental distress.

Character education is rooted in the principle that every individual possesses an intrinsic value and the capacity for moral growth. This growth is about nurturing virtues—qualities such as resilience, compassion, service, and integrity—that enable individuals to lead fulfilling lives and contribute positively to society. When character education is implemented within the school system, it operates under the compassionate premise that all students, irrespective of their mental health status, can benefit from developing a strong moral character. Character education stands not as a substitute but as a complementary force that can coexist with, and potentially enhance, traditional mental health and biomedical interventions. By emphasizing virtues, character education aims to cultivate protective factors that may fortify individuals against existential distress: distress that might be buffered against through the cultivation of meaning, purpose, aspired moral identity, and civic participation. However, character education will inevitably not be equally salutary for or protective against all kinds of mental illness; while many mental illnesses may be underpinned by both biological and existential factors, we must appreciate that the relative weighting of each may differ on a case‐by‐case basis. Moreover, character education recognizes the diverse experiences of individuals with mental health issues. It does not presume to “fix” an individual's mental illness through virtue cultivation but offers a supportive framework within which individuals might find greater meaning and strength in their lives.

To address mental health through the lens of character education is to adopt an approach that is inherently empathetic to the human condition. It is about providing students with the tools and understanding to engage with life's ethical dimensions while being sensitive to the complex nature of mental health. It is about teaching that challenges and suffering are part of the human experience and that enduring them can lead to profound personal growth and can help to provide young people with a deeper understanding of their own values and capabilities. In effect, character education strives to enrich the educational ecosystem with a dimension of moral and emotional depth, preparing students not just for academic success, but for the ethical and existential aspects of life. As a foundational element of schooling, we recognize that while we each have our unique struggles, the quest for moral growth and understanding is a shared human endeavor.

This work, situated at the intersection of clinical psychology, moral philosophy, and character education, exemplifies the interdisciplinary approach necessary to address the multifaceted issues of adolescence. By integrating insights from these diverse fields, this discussion contributes to a holistic understanding of how character education can help to protect mental health. This interdisciplinary perspective not only enriches the dialogue within each field but also offers a framework for developing educational interventions that are both morally and psychologically informed. Consequently, we hope that this paper makes a significant contribution to the special issue on interdisciplinary research on adolescence, highlighting the importance of collaborative efforts across disciplines to foster the well‐being and moral development of young people.

## CONFLICT OF INTEREST STATEMENT

The authors declare no known conflicts of interest. This article was partially proofread using Open AI's Chat GPT, but the intellectual content herein is our own.

## Data Availability

Data sharing not applicable to this article as no datasets were generated or analysed during the current study.
